# Functional organisation for verb generation in children with developmental language disorder

**DOI:** 10.1016/j.neuroimage.2020.117599

**Published:** 2021-02-01

**Authors:** Saloni Krishnan, Salomi S. Asaridou, Gabriel J. Cler, Harriet J. Smith, Hannah E. Willis, Máiréad P. Healy, Paul A. Thompson, Dorothy V.M. Bishop, Kate E. Watkins

**Affiliations:** a Department of Experimental Psychology & Wellcome Trust Centre for Integrative Neuroimaging, University of Oxford, Anna Watts Building, Radcliffe Observatory Quarter, Woodstock Road, Oxford, OX2 6GG, UK; b Department of Psychology, Royal Holloway, University of London, Egham Hill, Surrey TW20 0EX, UK; c MRC Cognition and Brain Sciences Unit, University of Cambridge, 15 Chaucer Road, Cambridge CB2 7EF, UK; d Nuffield Department of Clinical Neurosciences, John Radcliffe Hospital, Headley Way, Headington, Oxford OX3 9DU, UK; e Department of Psychology, University of Cambridge, Downing Street, Cambridge CB2 3EB, UK

**Keywords:** Functional neuroimaging, Specific language impairment, Paediatric, Language lateralisation

## Abstract

Developmental language disorder (DLD) is characterised by difficulties in learning one's native language for no apparent reason. These language difficulties occur in 7% of children and are known to limit future academic and social achievement. Our understanding of the brain abnormalities associated with DLD is limited. Here, we used a simple four-minute verb generation task (children saw a picture of an object and were instructed to say an action that goes with that object) to test children between the ages of 10–15 years (DLD N = 50, typically developing N = 67). We also tested 26 children with poor language ability who did not meet our criteria for DLD. Contrary to our registered predictions, we found that children with DLD did not have (i) reduced activity in language relevant regions such as the left inferior frontal cortex; (ii) dysfunctional striatal activity during overt production; or (iii) a reduction in left-lateralised activity in frontal cortex. Indeed, performance of this simple language task evoked activity in children with DLD in the same regions and to a similar level as in typically developing children. Consistent with previous reports, we found sub-threshold group differences in the left inferior frontal gyrus and caudate nuclei, but only when analysis was limited to a subsample of the DLD group (N = 14) who had the poorest performance on the task. Additionally, we used a two-factor model to capture variation in all children studied (N = 143) on a range of neuropsychological tests and found that these language and verbal memory factors correlated with activity in different brain regions. Our findings indicate a lack of support for some neurological models of atypical language learning, such as the procedural deficit hypothesis or the atypical lateralization hypothesis, at least when using simple language tasks that children can perform. These results also emphasise the importance of controlling for and monitoring task performance.

## Highlights

• We scanned the largest cohort of children with developmental language disorder to date.
• Our pre-registered predictions were not upheld.
• Children with DLD who accurately performed the verb generation task showed no functional abnormality.
• Proficiency on language and verbal memory factors correlated positively with activity in distinct brain areas.
• Functional neural differences emerged for a subset of DLD who performed the task poorly.

## Functional organisation for verb generation in children with developmental language disorder

Children with developmental language disorder (DLD) struggle to learn their native language for no apparent reason. It is a common but under-recognised condition (Bishop, 2014). The prevalence of DLD is estimated to be 7% at school entry (Norbury et al., 2016). As with other better-known neurodevelopmental disorders, such as autism spectrum disorder (ASD) and attention deficit hyperactivity disorder (ADHD), there are no sharp dividing lines between typical development and disorder, and the definition encompasses a range of language problems (Bishop et al., 2016). One reason for lack of recognition could be the inconsistent terminology and criteria used: a recent consensus study agreed on the term DLD to replace other terms, including Specific Language Impairment (SLI) (Bishop et al., 2017). In terms of aetiology, DLD is thought to be a complex, multifactorial disorder caused by a combination of many genetic influences each of small effect, interacting with environmental factors (Bishop, 2006).

Relatively little is known about the neurological basis of developmental language disorders. An early study by Jernigan et al (1991) found that these disorders do not usually result from gross lesions of the brain, but differences in relative sizes of different brain regions have been described. Classically, studies have suggested that the structure and activity in language-relevant regions such as the inferior frontal gyrus and the superior temporal gyrus differ in DLD and typically developing children. A few studies also indicated structural abnormalities and dysfunctional activity within the striatum (Badcock et al., 2012; Lee et al., 2013; Watkins et al., 2002b), a structure we hypothesized is important for language learning (Krishnan et al., 2016). However, a recent systematic review highlighted the small number of studies probing structural or functional abnormalities in DLD, and noted that each study involved very small numbers of participants (Mayes et al., 2015); as a result, findings across studies are inconsistent.

One long-standing theory maintains that DLD and other neurodevelopmental disorders involving speech and language (e.g. dyslexia, stuttering) are associated with a failure to establish normal patterns of cerebral lateralisation for language (see Bishop, 1990, for review). More recent studies using a range of brain imaging methods have lent some support to this view (Bishop, 2013). Using functional MRI to measure laterality indices across a battery of language tasks, children with DLD showed a lack of left lateralisation in core language areas (de Guibert et al., 2011). Our own preliminary work in a small sample of eight children with DLD also showed reduced left lateralisation in frontal lobe activity on an auditory responsive naming task (Badcock et al., 2012). However, a recent large study using functional transcranial Doppler sonography (fTCD) to assess language lateralisation in 263 children found rates of atypical lateralisation in the DLD group to be no different to those in the typically developing group (Wilson and Bishop, 2018). This led the authors to speculate that previous findings of association could be false positives arising from a literature characterised by small sample sizes and analytic flexibility. Alternatively, fTCD might be insensitive to aspects of language laterality measured with functional MRI: fTCD is sensitive and reliable in detecting task-related changes in blood flow in the middle cerebral artery, but it does not give any information about localisation of activation within the hemisphere.

Discrepancies in findings across functional studies could also be explained by the range of paradigms used. Of the small number of fMRI studies comparing DLD with typically developing groups, each used different tasks and focused on different abnormalities. When listening to nonwords and words, five individuals with DLD showed weaker activity in the superior temporal gyrus bilaterally relative to age-matched controls (Hugdahl et al., 2004). In another task (Ellis-Weismer et al., 2005), participants responded to auditory questions (encoding phase) and subsequently were quizzed about whether they had heard the question before (recognition phase). In the encoding phase, those with DLD (N = 8) showed reduced activity in the left precentral sulcus and parietal cortex, whereas in the recognition phase, they showed abnormal decreases in the left inferior frontal gyrus. In a task-switching paradigm designed to examine executive function, those with DLD (N = 4) had greater activity than controls in the left superior temporal gyrus (Dibbets et al., 2006). In our own work, we used an auditory responsive naming task, which had fewer meta-linguistic demands (Badcock et al., 2012). Participants heard a three-word definition and covertly generated a corresponding word. Those with DLD (N = 8) showed reduced activity in the left inferior frontal cortex, right putamen, and the superior temporal gyrus bilaterally. Most recently, using a functional connectivity analysis of brain activity during an implicit word segmentation task, adults with DLD (N = 16) showed hyperactivity in the left inferior frontal gyrus, superior temporal gyrus, and supramarginal gyrus (Plante et al., 2017). Each of the functional paradigms described above is associated with a different pattern of neural activation, perhaps leading to differential power for picking up group differences. In addition, dysfunction during task is characterized as either over- or underactivity, sometimes in the same brain region. An important consideration when testing two groups is to minimise performance-related differences on tasks (Brown et al., 2005; Schlaggar et al., 2002). If children with DLD do not perform the task in the scanner at the same level as the comparison children, then any differences in activation might just be a consequence of this poor performance, rather than telling us anything about the cause of language disorder. Many of the studies above (including our own) used covert tasks, making it difficult to assess whether those with DLD were performing the task at the same level. In other cases, such as in the tasks making demands on executive functioning, those with DLD are known to perform poorly (Gooch et al., 2016; Kapa and Plante, 2015).

For the current study, we identified verb generation as a functional imaging task that would be suitable for probing brain activity for language processing in children with different levels of language ability. A typical verb generation task involves identifying a picture, searching the mental lexicon for a plausible action that would relate to the picture, retrieving that action word, phonological assembly, and execution of the articulatory commands for overt speech production of the word. In adults, a consistent pattern of brain activity is observed when this task is performed, namely robust activation of the posterior part of the left inferior frontal gyrus (Petersen et al., 1988; Thompson-Schill et al., 1997). Other regions that are commonly activated are regions in the posterior peri-Sylvian cortex, including the supramarginal gyrus, posterior superior temporal gyrus and sulcus, the anterior cingulate cortex, and the supplementary motor area; these areas are often activated bilaterally, although with the midline structures this is difficult to discern. Verb generation tasks have also been successfully used with children (Brown et al., 2005; Holland et al., 2001; Schapiro et al., 2004; Schlaggar et al., 2002; Szaflarski et al., 2006, 2005). Covert versions of this task show how brain activity for language processing changes with age, but as noted above preclude determination of performance-related changes (Holland et al., 2001; Karunanayaka et al., 2010; Szaflarski et al., 2006, 2005). Behaviourally, we know that children with DLD can perform verb generation tasks (Norbury et al., 2001). This, together with the fact that the task reliably produces lateralised activation in the prefrontal cortex, make it well-suited to our purposes. In addition, this task can be completed overtly, making it possible to track any performance-related issues.

Where in the brain might we expect to see neural differences between those who are typically developing, and those with speech and language disorders, on a verb generation task? Overt verb generation has been studied in affected members of the KE family, who have a mutation in the gene *FOXP2* and a behavioural profile consistent with DLD (Watkins et al., 2002a). Although the previous fMRI study (Liégeois et al., 2003) compared only a very small number of participants (five affected members to five unaffected members), it allows us to make predictions about regions where we would expect to see differences between those with DLD and those without. Results revealed a diffuse pattern of bilateral activity in affected relative to unaffected family members, with significantly reduced activity in the pars triangularis of the left inferior frontal gyrus and in the putamen bilaterally (Liégeois et al., 2003). The posterior part of the left inferior frontal gyrus is a key region for language processing; tasks engaging in lexical search and retrieval activate this region robustly, with separable loci for phonological processing in pars opercularis and semantic processing in pars triangularis (Gough et al., 2005). The putamen is likely to play a role in the smooth execution of the complex sequential and simultaneous movements that are necessary for speech. We have previously hypothesised that subcortical brain regions involved in speech and language learning, such as the striatum, may be dysfunctional in DLD (Krishnan et al., 2016).

On the basis of these previous observations, we generated the hypotheses below. Our aim was to collect data from at least 45 children with DLD aged 10–15 years and 45 children who are typically developing and matched for age and sex. The minimum sample size of 45 was chosen based on a power analysis (see methods).

Hypothesis 1: children with DLD show reduced activity relative to typically developing children in the frontostriatal network, specifically in the pars triangularis of the left inferior frontal gyrus (BA45) (Hypothesis 1a) and in both the left and right putamen (Hypothesis 1b and c); the two groups will not differ in terms of task-related activity in a brain region outside this network, namely the right lateral occipital cortex (Hypothesis 1d).

Hypothesis 2: children with DLD show reduced left lateralisation in the frontal lobe during verb generation (Hypothesis 2a). Such a reduction could be due to failure to reliably activate either hemisphere above noise levels, reduced activity in the left hemisphere or increased activity in the right hemisphere. On the basis of previous work, we predict that any reduction in laterality observed will be due to a failure to reliably activate either hemisphere above noise levels (Hypothesis 2b).

## Methods

### Ethics

This study was approved by the Medical Sciences Interdivisional Research Ethics Committee at the University of Oxford (R55835/RE002). Before enrolling participants in the study, we obtained written informed consent from parents/guardians, and written assent from children.

### Pre-registration

The pre-registered Stage 1 manuscript is available at https://osf.io/6g247.

### Participants

We recruited participants between the ages of 10;0 to 15;11(years;months) across a range of language abilities for this study. Some children participated in the SCALES study (Norbury et al., 2016), the Wellcome Reading and Language Project (Snowling et al., 2015), and the OSCCI Twins Study (Wilson and Bishop, 2018). In addition, we recruited participants in this age range from schools for children with language learning difficulty, as well as advertising through organisations that conducted outreach with those with language problems (such as ICAN, Afasic, RADLD) and dyslexia (such as the British Dyslexia Association). We primarily recruited typically developing participants from local schools and schools participating in university outreach programs.

Our inclusion criteria for all participants were: (i) normal hearing (defined as passing audiometric screening at 25 dB at 500 Hz, 1000 Hz, and 2000 Hz, in the better ear); (ii) a nonverbal IQ > 70 (assessed using the WISC-IV Matrix Reasoning and Block Design Tests – Wechsler, 2004); and (iii) having grown up in the UK speaking English. Children were not recruited if they met any of the following exclusion criteria: (i) a diagnosis of another developmental disorder such as Down syndrome or Williams syndrome; (ii) a history of neurological impairments or neurological disorders such as epilepsy; (iii) a diagnosis of ASD or ADHD; (iv) a score above 7 (i.e. in the clinical range) on the hyperactivity subscale of the Strengths and Difficulties Questionnaire (SDQ; Goodman, 1997); (v) a score above 15 on the Social Communication Questionnaire – Lifetime (Rutter et al., 2003); (vi) a contraindication to MRI. Children were not excluded on the basis of their handedness or if they spoke multiple languages.

Participants were categorised as typically developing if they had no history of speech and language problems, and if no more than one standardized language test score was 1 SD or more below normative mean (see (Barry et al., 2007), for rationale for this criterion, and see below for tests included as part of our assessment).

Participants were categorised as having DLD if they presented with a history of speech and language problems and scored 1 SD or more below the normative mean on two or more standardised tests of language ability (see below).

We predicted that some participants would have a history of speech and language (HSL) problems, but would fail to meet our DLD criteria (i.e., 1 SD or more below the mean criterion on two language tests). These cases (HSL participants) are included in analyses using quantitative language phenotypes, but not in group comparisons. Similarly, we predicted that children who are considered typically developing could score 1SD or more below the mean on two standardized language tests but have no history of speech and language problems. We decided to exclude such children from our analyses (see Results, participant selection, for further details regarding exclusions).

We also excluded data from participants who moved excessively during the functional run or in whom we could not obtain a T1-image for registration. Excessive movement was defined if one or both of the following criteria are met: (i) the average absolute motion correction applied using MCFLIRT (part of FSL, see below for more details) to each volume to bring it into registration with the reference volume > 2.4 mm (the dimension of one imaging voxel); (ii) the number of outliers detected by fsl_motion_outliers (a tool that detects volumes in the time-series that have been corrupted by large motion and that cannot be fixed using linear motion parameter regression methods) exceeds 75 volumes out of the 300 acquired during the task (i.e. 25%).

Where participants failed to complete behavioural testing, we imputed data for the purposes of factor analyses if no more than two test scores were missing (using full information maximum likelihood in the analysis, see Results section for more details). Participants who failed to complete three or more behavioural tests were excluded from our analyses.

### Neuropsychological battery

In addition to the screening measures described above (SDQ and SCQ), participants completed a neuropsychological battery to evaluate their language skills, nonverbal reasoning ability, handedness, and motor dexterity/co-ordination. The entire battery of tests took less than 2.5 hours to administer.

#### Language tests

Participants’ language ability was assessed using tests of grammar, narrative and vocabulary; a score on these tests of 1 SD or more below the mean was used to categorise children with DLD. Grammatical comprehension was assessed using the Test for Reception of Grammar – 2 or its electronic counterpart (TROG-E, Bishop, 2005). This is a multiple-choice sentence comprehension test. Expressive grammar was evaluated using the Recalling Sentences subtest of the Clinical Evaluation of Language Fundamentals – 4^th^ Edition (CELF-4; Semel et al., 2004), which involves repeating sentences verbatim. This process draws on processing, analysis, and reconstruction of the meaning of sentences using the child's language and memory systems, and consequently provides a window into their grammatical ability. Children's narrative production and comprehension was assessed using the Expression, Reception and Recall of Narrative Instrument (ERNNI; Bishop, 2004). Receptive and expressive vocabulary were assessed using the Receptive One-Word Picture Vocabulary Test – 4th Edition (ROWPVT-4; Martin and Brownell, 2011a) and Expressive One-Word Picture Vocabulary Test – 4th Edition (EOWPVT-4; Martin and Brownell, 2011b) respectively.

#### Reading tests

We obtained measures of phonological decoding and sight word reading efficiency using the Test Of Word Reading Efficiency (TOWRE; Torgesen et al., 1999); scores on this test were not used to assign children to the DLD group.

#### Intelligence tests

To assess nonverbal reasoning ability, participants completed the block design, matrix reasoning, and coding subtests of the Wechsler Intelligence Scale for Children - 4^th^ Edition (WISC-IV; Wechsler, 2004). Only performance on the block design and matrix reasoning sub-tests was used to calculate non-verbal IQ.

#### Memory tests

We assessed short-term and working memory using the forward and backward digit span subtests from the Children's Memory Scale (CMS; Cohen, 1997). We also used the word lists subtest from the CMS to assess recall of items presented multiple times. For the tests listed above, raw scores were converted into age-scaled scores using published norms. To assess phonological short-term memory and the ability to articulate unfamiliar sequences, we used a nonword repetition test that has been previously used by the SCALES project (Norbury et al., 2016) and the Wellcome Language and Reading Project (Snowling et al., 2015). Raw scores from this test were used as no published norms are available.

#### Motor tests

The nonword repetition test can also be considered a test of articulatory sequencing (Krishnan et al., 2013, Krishnan et al., 2017). Oromotor coordination was also assessed using the oromotor sequences sub-test of the NEuroPSYchology (NEPSY) test battery (Korkman et al., 1998); raw scores are reported. Handedness was assessed by asking participants to self-report their preferred hand for writing. Relative hand skill was assessed using scores on the Purdue Pegboard (Brookman et al., 2013; Tiffin, 1968) and converted into age- and gender-scaled norms based on published norms in the manual.

### Summary measures

Planned analyses to assess associations between brain measures and language development used factor scores, which enhanced the reliability of the measure, and minimised the number of multiple comparisons in statistical tests.

The measures from the language and memory tests described in sections (i) and (iv) above were entered into a factor analysis to identify the best weighted combination of measures to give a language factor score, and a memory factor score. The approach we adopted was E-CFA (Brown, 2006), implemented in lavaan (Rosseel, 2012) in the R programming language (R Core Team, 2020). E-CFA is a hybrid exploratory-confirmatory approach to factor analysis where a model is specified with an ‘anchor’ measure or two anchor measures. No cross-loadings were specified for each factor. The model allowed other measures to load on both factors, with paths being dropped if they did not improve model fit. As anchor measures, we used the list learning standard score from the CMS for the memory factor, and expressive vocabulary for the language factor. We proposed testing the model with two factors against a single factor model; if the latter provided an equally good fit (as assessed by Bayesian Information Criteria [BIC]), we proposed to conduct analyses using one rather than two outcome measures.

## Functional task

### Materials

Verb generation norms were recently reported for a subset of Snodgrass and Vanderwart (Snodgrass and Vanderwart, 1980) pictures by Kurland and colleagues (2014). We chose twenty-four pictures on the basis of their high verb agreement across participants (>80%), (see https://osf.io/k5bfs/?view_only=905d3d0b244e4ab5883dc56e180ad299). However, perfect verb agreement was not obtained for any of the pictures. Pictures were sourced from the Rossion and Pourtois dataset (Rossion and Pourtois, 2004), which are colourful versions of the Snodgrass and Vanderwart pictures, rather than black and white line drawings.

### Design

The experimental design comprised eight alternating blocks of verb generation and rest. Each block lasted 30 s and the entire run was four minutes long. During the four verb generation blocks, participants were presented with pictures and asked to overtly generate a verb associated with the picture. For instance, participants could say “throw” if shown a picture of a ball. Each trial lasted five seconds; participants completed six trials in every verb generation block. Verbal responses for each picture were audio recorded using a noise cancelling microphone for later scoring. In considering task design, we had to decide on which baseline to use. Although we and others have noted the value of including multiple baselines in developmental research (Krishnan et al., 2015), each baseline condition that is added increases the duration of the task. This issue can lead to compromised data quality when working with children, especially those with language problems. Accordingly, we decided to use ‘rest’ as the baseline condition, on the grounds that this should be effective in allowing us to capture the largest neural differences between children with language disorders and those who are typically developing. During the rest blocks, participants were asked to lie still and relax. A white screen was displayed for the duration of the rest block. The task was coded using PsychoPy v1.84.2; the code is available at https://osf.io/k5bfs/.

### MRI acquisition

MR data were collected with a 3T Siemens Prisma scanner with a 32-channel head coil. Participants wore noise-cancelling headphones (Optoacoustics OptoActive II Active Noise Cancelling Headphones) and overt responses were recorded with a noise-cancelling microphone (Optoacoustics FOMRI-III microphone). Foam padding was placed around the head for comfort and to restrict movement; the headphones were held in place with inflatable pads.

Functional scan parameters were matched to the ABCD study (Casey et al., 2018). Specifically, fMRI data consisted of 325 volumes of 60 *T*_2_*-weighted echo-planar image (EPI) slices (repetition time [TR] 800 ms, echo time [TE] 30 ms, flip angle 52^o^, field of view 90 × 90 mm, with multiband acceleration factor of 6), yielding a 2.4 × 2.4 × 2.4 mm resolution. Total acquisition time was 4 min and 33 s. The first 25 volumes were discarded, as these were acquired when the noise cancelling algorithm was learning the scan sequence. Noise cancellation was applied during acquisition of the following 300 volumes. We also collected a B0 field map to help correct distortions. For registration purposes, a *T*_1_-weighted MPRAGE scan (magnetization prepared low angle spoiled gradient echo, TR 1900 ms, TE 3.97 ms, flip angle 8^o^, field of view 208 × 256 × 256 mm) was acquired during the scanning session with 1 mm in-plane resolution and 1 mm slice thickness. The acquisition of the *T*_1_-weighted image took 5 mins and 30 s.

### Procedure

A verb generation task was chosen as it is engaging for children and easy for them to comply with. Prior to the scan, the experimenter verbally explained the task to the child (while outside the scanner). Children were told to generate an action verb every time they saw a picture appear on screen. They were instructed that there would be occasions when nothing appeared on screen, and they should relax while staying as still as possible. Children practised the task outside the scanner using stimuli different to those they encountered in the scanner. Participants were also told to move minimally while they overtly produced speech and were given feedback on their level of movement before scanning started (when lying on the scanner bed with the headphones and microphone in place).

In the scanner, participants completed a set of scans, including a resting-state scan, another functional task, and structural scans (multi-parameter-mapping and diffusion weighted imaging). Participants were reminded of the task instructions prior to the start of the verb generation task. For the structural scans, participants were given a choice of movies they to watch. They were reminded to lie still while they watched the movie.

## Imaging data analysis

### Preprocessing

FMRI data processing was carried out using FEAT (FMRI Expert Analysis Tool) Version 6.00, part of FSL (FMRIB's Software Library, www.fmrib.ox.ac.uk/fsl). Preprocessing of all data followed standard procedures consisting of identification of motion outliers using fsl_motion_outliers, motion correction through realignment to a reference volume acquired prior to the task (MCFLIRT), skull stripping using BET (Brain Extraction Tool), spatial smoothing using a 5 mm full-width at half-maximum Gaussian kernel, and high-pass temporal filtering with a cut-off of 60 s. To improve image registration with the structural scan, fieldmaps were used to unwarp the functional data employing PRELUDE (Phase Region Expanding Labeller for Unwrapping Discrete Estimates) and FUGUE (FMRIB's Utility for Geometrically Unwarping EPI; Jenkinson 2003). EPIs were registered using boundary-based registration (Greve & Fischl, 2009) to the individual participant's T1-weighted structural image, which in turn were registered to the MNI-152 template using FNIRT (FMRIB's Non-linear Image Registration Tool).

### First-level analysis

For each participant, task-based statistical parametric maps were computed for the contrast of the verb generation condition to the rest baseline using the general linear model (GLM) based on the experimental time course convolved with a double-gamma function and its temporal derivatives. Image volumes that were outliers in terms of motion (determined for each functional scan using fsl_motion_outliers), and the six motion correction parameters (translations and rotations in x, y and z) were included as covariates of no interest in the analyses.

### Planned statistical analyses

(i) Hypothesis 1: children with DLD show reduced activity relative to typically developing children in the frontostriatal network, specifically in the pars triangularis of the left inferior frontal gyrus (BA45) (Hypothesis 1a) and in both the left and right putamen (Hypothesis 1b and c); the two groups will not differ in terms of task-related activity in a brain region outside this network, namely the right lateral occipital cortex (Hypothesis 1d).

We used Featquery to extract % BOLD signal for verb generation > rest in the left inferior frontal gyrus and the putamen bilaterally in each participant. We used % BOLD signal from the right lateral occipital cortex, which shows a response to the picture stimulus presented as a control region, in which we did not expect to see a difference between groups. These four ROIs were created using the probabilistic masks for left inferior frontal gyrus (pars triangularis), left and right putamen, and right lateral occipital cortex, inferior division from the Harvard-Oxford cortical structural atlas available in FSL; each mask was thresholded at > 30% of the atlas participants.

Statistical inferences used a null hypothesis significance testing approach, with alpha set at .05. Hypotheses 1a-c were tested using independent samples t-tests between the groups of DLD and typically developing children for data from the left pars triangularis, left putamen, and right putamen, respectively. These hypotheses were directional, in that we predicted that those with DLD will have reduced activity relative to the group of typically developing children. Consequently, we used one-tailed t-tests. For hypothesis 1d, we compared data from the right lateral occipital cortex in the two groups using an independent t-test but as we did not predict a significant difference in either direction we used a two-tailed test. We corrected the alpha for the number of tests (four; alpha < .0125).

In a secondary set of regression analyses, we used the language and memory factors as predictors of activity in these four regions. This allowed us to evaluate if there was a continuous relationship between language/memory ability and activity in these four regions. We were able to include more participants in the second analysis, as some children with poor language ability did not meet our criteria for DLD. We conducted four stepwise regression analyses using BOLD activity in each ROI as the dependent variable, and the language and memory factors as independent variables. We controlled for age and task performance in these models by entering them as control variables. For activity in the left inferior frontal gyrus, the left putamen, and the right putamen, our hypotheses were directional. We predicted that those with lower language and memory ability would have reduced activity in these regions (akin to hypotheses 1a-c). For activity in the right lateral occipital cortex, we expected to see no association with language and memory scores (akin to hypothesis 1d). Again, we corrected alphas for the number of tests (four, alpha <.0125).

(i) Hypothesis 2: children with DLD show reduced left lateralisation in the frontal lobe during verb generation (Hypothesis 2a). Such a reduction could be due to failure to activate either hemisphere above noise levels, reduced activity in the left hemisphere or increased activity in the right hemisphere. On the basis of previous work, we predict that any reduction in laterality observed will be due to a failure to activate either hemisphere above noise levels (Hypothesis 2b).

Lateralisation of functional brain activity during verb generation > rest was measured using lateralisation indices, which were calculated using the LI toolbox (Wilke and Lidzba, 2007), run in SPM12. This toolbox employed a weighted-bootstrapping algorithm to generate threshold-free LI values. LIs were iteratively calculated at increasing thresholds to produce a laterality curve. The LI was calculated from a weighted mean of 20 equally sized intervals from Z = 0 to the maximum value in the masked image (Wilke and Schmithorst, 2006). This approach reduces the threshold-dependent nature of calculating LI (Bradshaw et al., 2017). We calculated LIs for the frontal lobes using the standard templates included in the toolbox while excluding the medial walls 5 mm either side of the centre of image. The laterality index formula is LI = (L − R)/(L + R). Positive values indicate left lateralisation and negative values indicate right lateralisation. Previous studies have adopted the convention of considering values between 0.2 and -0.2 as indicative of bilateral processing with values outside this range being indicative of left- or right-lateralised processing (Wilke et al., 2005; 2006). Participants were categorised as left- or right-lateralised or bilateral using this convention.

To test hypothesis 2a, we first used chi-squared analyses to determine whether there was a linear-by-linear association of these three categories with DLD status. If insufficient numbers (< 5 participants) occurred within cells, we combined the LI categories into typical (left-lateralised) and atypical (right-lateralised or bilateral). Our hypothesis 2a was directional in that we expected reduced LI in the DLD group so we used a one-tailed test. As with the ROI-based analysis above, we conducted a secondary analysis exploring the relationship between LIs and the two composite language and memory indices across the whole population of children scanned using nonparametric correlational analysis (Spearman's rho).

To address Hypothesis 2b, that reduced LI is due to a failure to activate either hemisphere above noise levels, we planned to compare the number of voxels surviving a threshold of Z > 4.42 (*p* < .000005 uncorrected) for the verb generation > rest contrast in each frontal lobe mask used in the LI calculation in children with DLD relative to typically developing children. We predicted that the number of voxels reliably activated across the frontal lobes would be significantly lower in both hemispheres in children with DLD relative to the control group.

Analyses were conducted in R (R Core Team, 2020), and plots were generated using the rainclouds plot package (Allen et al., 2019). 3D brain renderings were created using connectome workbench visualisation software (Marcus et al., 2011). Slices were rendered using MRIcroGL (Rorden et al., 2007).

### Exploratory analyses

In addition to the ROI analysis described above, we planned two models at the whole-brain level, 1) a between-group comparison of those that are typically developing and those with DLD, and 2) a correlation analysis between language ability (using the summary measures for language and memory functions described above) and neural activity for the verb generation > rest contrast. The use of these models allows us to assess if language variation is linked to regions beyond those predicted and assessed in the ROI analysis. Group averages, differences between groups, and the correlation between language ability and activity, for the contrast of the Verb Generation condition to the “rest” baseline were calculated at a second-level analysis using FMRIB's Local Analysis of Mixed Effects (FLAME) stage 1 (Woolrich et al., 2004). Variance for the two groups was estimated separately as we expected it to differ. Statistical maps were cluster-thresholded at Z > 3.1 and clusters reported that survived a statistical test for extent (*p* < .05, family-wise-error corrected).

We complemented the whole-brain averages and group differences with measures of inter-subject variability by generating probabilistic overlap maps. Overlap maps are used to visualise consistency in patterns of activation and can be considered as a measures of reliability across participants (Specht et al., 2003). For the verb generation > rest contrast in each participant, z-statistics were thresholded voxel-wise at Z > 4.42 (*p* < .000005 uncorrected and registered to MNI standard space. Resulting images were binarized by assigning each voxel a 1 or 0 depending on whether the voxel exceeded the statistical voxel-wise threshold or not. These binary maps were summed across DLD and typically developing participants and divided by the total in each group, to obtain an image showing the spatial consistency in activation across participants in each group separately (i.e. the percentage of each group who activated each voxel above threshold).

### Justification of sample size

We had funding to collect 160 datasets over the course of this study, and we planned to recruit 80 children with developmental language disorder or poor language ability. As we noted during the stage 1 submission, some datasets were collected prior to in-principle acceptance of this report. As stated in the submission, given that we did not intend to change our protocol mid-way through our study, we proposed to use these data in our analyses, but would note these numbers. Ten datasets were presented as pilot data during the review process; we have excluded these data. Forty additional datasets included in the report (38 TD, 1 DLD, 1 HSL) were acquired before we received the in-principle acceptance.

An indicative power analysis was run using our previous data (Badcock et al., 2012), although the paradigm used in this work was auditory responsive naming, not verb generation. We constructed masks of the left inferior frontal gyrus, pars triangularis, and left and right putamen as described above. We estimated mean activity for both typically developing children and those with DLD in these regions of interest. This analysis indicated that to detect a significant group difference in these regions at an alpha level of *p* < .05 (one-tailed) with 80% power, we would need at least 29 participants in each group (the smallest effect size was seen in the left inferior frontal gyrus, pars triangularis, d = 0.67). Using an alpha level of *p* < .0125 (Bonferroni-corrected for 4 ROIs) with 80% power, we would need at least 44 participants in each group. This analysis suggests that our proposed sample of 160 participants, or 80 participants in each group, should be sufficient to test our hypotheses. We aimed to achieve a minimum of 45 participants per group.

## Results

### Participant selection

We recruited and tested 175 children. Data from 10 TD participants were excluded because these contributed pilot data for the Stage 1 submission. Another three did not complete the MRI session and another 1 did not complete behavioural testing. Six were excluded because they were subsequently found not to meet our inclusion criteria (3 did not grow up in the UK speaking English, 3 failed our non-verbal IQ criteria). One TD child was excluded because they had scores of 1SD or more below the mean on two standardized tests of language (our criteria for DLD), another due to an incidental finding of unknown clinical significance and a third due to a technical fault during scan acquisition. Of the remaining 152 eligible datasets, data from 5 participants (4 DLD, 1 HSL) were excluded because they failed our motion criteria. Additionally, 4 DLD children did not pass our accuracy criterion of 75% on the in-scanner verb generation task (see Fig. 2). Our sample size was consequently 50 children with DLD and 67 TD children. The HSL group included 24 children who were recruited as DLD, but testing showed that they did not fully meet the DLD criteria, and another 2 children initially recruited as TD in whom histories of speech and language problems were subsequently reported. Data from the children in the HSL group were included only in the analyses which looked at the relationship between the neuropsychological factors and task-evoked responses in the imaging data across the whole population. Descriptive data characterising our three groups (DLD, HSL, TD) are shown in Table 1.

**Table 1:** Descriptive Data for the Typically Developing (TD), Developmental Language Disorder (DLD), and History of Speech and Language Problems (HSL) groups. Means are shown below, with standard deviations in parentheses. The last column shows whether there were significant group differences when using t-tests or Chi-squares (*p* < .05), no correction for multiple comparisons is applied. Unless otherwise specified, tests without a superscript denote standard scores with a mean of 100 and SD of 15. ^1^ - Scaled scores with a mean of 10 and SD of 3. ^2^ - Raw scores are shown (note that the maximum possible oromotor sequencing score = 70, maximum possible nonword repetition score = 30).

	TD	DLD		HSL	Group differences
N	67	50		26	
Age (years)	12.1 (1.7)	12.0 (1.7)		11.9 (1.7)	None
Gender	38M:29F	35M:15F		21M:5F	None
Handedness	58R:9L	44R:6L		23R:3L	None
***Language Scores***					
TROG-E	105.4 (8.3)		83.0 (13.9)	98.2 (8.1)	TD>HSL>DLD
CELF Recalling Sentences^1^	11.9 (2.2)		5.2 (2.6)	9.0 (3.0)	TD>HSL>DLD
ROWPVT	127.8 (16.1)		100.5 (15.7)	122.4 (15.8)	TD+HSL>DLD
EOWPVT	118.4 (14.9)		91.9 (13.1)	108.1 (13.8)	TD>HSL>DLD
ERRNI Comprehension	106.3 (13.4)		92.4 (15.1)	101.8 (10.6)	TD+HSL>DLD
ERRNI Initial Recall	99.7 (13.1)		84.3 (12.7)	96.8 (11.0)	TD+HSL>DLD
ERRNI Delayed Recall	104.6 (12.0)		84.3 (12.8)	99.8 (10.2)	TD+HSL>DLD
***Non-verbal IQ***					
Matrix Reasoning^1^	11.1 (2.7)		7.6 (3.2)	10.0 (2.7)	TD+HSL>DLD
Block Design^1^	13.3 (2.0)		9.9 (3.2)	12.8 (2.2)	TD+HSL>DLD
***Reading Tests***					
Sight Word Reading Efficiency	106.4 (11.5)		82.9 (13.4)	89.3 (14.3)	TD>HSL+DLD
Phonological Decoding	111.8 (14.0)		81.5 (15.3)	87.3 (13.4)	TD>HSL+DLD
***Memory Tests***					
Nonword repetition^2^	26.3 (2.4)		18.1 (5.3)	22.8 (3.8)	TD>HSL>DLD
CMS Initial Recall^1^	10.3 (2.9)		6.2 (2.8)	8.2 (2.9)	TD>HSL>DLD
CMS Delayed Recall^1^	10.2 (3.1)		7.3 (3.3)	9.6 (3.1)	TD+HSL>DLD
CMS Delayed Recognition^1^	8.4 (3.3)		6.8 (3.7)	7.3 (3.0)	TD>DLD
Digit Span Forward^1^	11.5 (2.7)		6.1 (3.1)	7.5 (3.2)	TD>HSL+DLD
Digit Span Backward^1^	11.8 (2.5)		7.5 (3.3)	8.6 (3.0)	TD>HSL+DLD
***Motor Scores***					
Oromotor sequencing^2^	60.9 (7.3)		42.2 (10.6)	53.3 (10.2)	TD>HSL>DLD
Pegs moved with dominant hand (z-score)	-0.4 (0.9)		-1.6 (1.2)	-0.9 (0.8)	TD>HSL>DLD
Pegs moved with non-dominant hand (z-score)	0.0 (1.0)		-1.2 (1.3)	-0.7 (0.9)	TD>HSL>DLD
Mean difference of pegs moved (dominant – non-dominant hand)^2^	0.6 (1.5)		0.6 (1.4)	0.6 (1.4)	None

### Summary measures

Exploratory data analysis revealed only minor deviations from normality in the distributions of the measures that we proposed summarising for our factor analysis. Given that some measures were standard scores, and two were raw scores (nonword repetition and oromotor sequencing), we also assessed possible correlations of raw scores with age. These were not significant in either the typically developing or language disordered children. We computed summary measures using our two pre-registered models. We used maximum likelihood estimation, with full information maximum likelihood (FIML) for the missing data. We standardized the latent factors, allowing free estimation of all factor loadings. All R code for the analysis is available on OSF (https://doi.org/10.17605/OSF.IO/2WPX5).

**Fig. 1:**
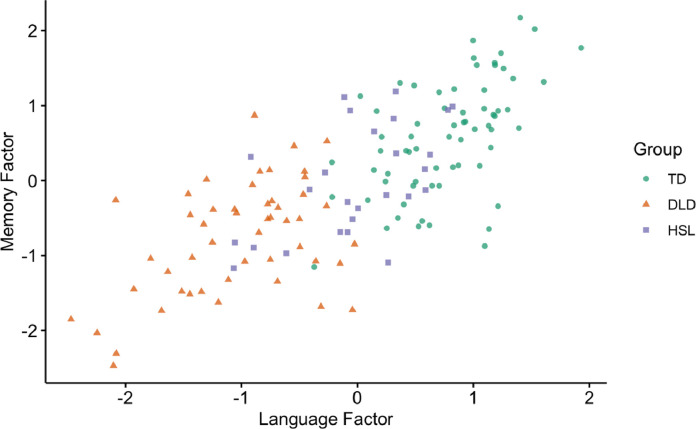
The relationship between language and memory proficiency scores in our sample is illustrated here. Language factor scores are plotted on the X axis, and memory factor scores are plotted on the Y axis. Colour and shape depicts group membership (TD – green circles, DLD – orange triangles, HSL – purple squares).

Statistical comparisons indicated that the two-factor model fit the data significantly better than the single-factor model, χ^2^(13) = 172.11, *p* < .001, however neither pre-registered model was a good fit (TLI < .795, CFI < .856, SRMR > .065). We consequently examined the modification indices of both models to improve model fits. For both models, this indicated that expressive and receptive vocabulary scores, as well as the two narrative production measures (ERRNI initial and delayed recall) were strongly correlated with each other, with modification indices of > 30. These correlations were subsequently modelled for both the single and two-factor models. In addition, for the single-factor model, modification indices suggested strong correlations between the memory scores; but these were not modelled as this was effectively what the two-factor model captured. The modified two-factor model had an acceptable fit, with a TLI of .93, CFI = .952, SRMR = .046, and RMSEA of .082, 90% CI (.06–.104). Again, statistical comparisons showed that the modified two-factor model provided a significantly better fit than the modified single-factor model, χ^2^(13) = 172.19, *p* < .001. The BIC value for the modified single-factor model was 12,896.92, whereas BIC for the modified two-factor model was 12,789.24, confirming the significant improvement gained from the two-factor model when also allowing for model complexity. We consequently derived language and memory proficiency scores on the basis of the modified two-factor model. The relationship between language and memory proficiency scores, and mapping to group membership, is shown in Fig. 1.

### Verb generation task performance

**Fig. 2:**
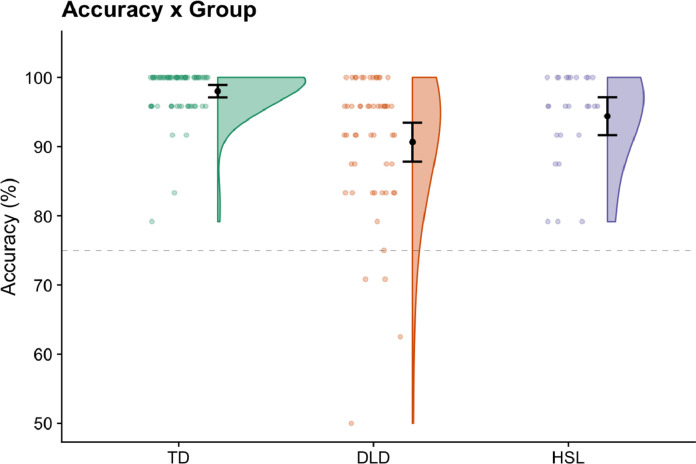
Accuracy scores for verb generation in typically developing (TD) children (in green), those with developmental language disorder or DLD (orange), and those with a history of speech and language disorder or HSL (in purple) are depicted using violin plots. Plots show mean +/- 95% confidence intervals. Individual data is shown alongside; data are jittered along the X axis for visualisation purposes. Dotted line indicates our performance cut off for inclusion in analyses. The four individuals below the cut off were only retained for the performance sub-group analyses (see text and later figures).

Responses made during the verb generation were manually marked as accurate or inaccurate at the scanner, and these were later verified using audio recordings. Responses were coded as accurate is an appropriate verb was generated (e.g. [image of kite]-fly). They were marked inaccurate if there was no response, a generic verb (e.g., [image of ruler]-*use*), a noun (e.g., [image of bell]-*noise*), or a verb that did not make sense (e.g., [image of kite]-*create*). Typically developing children were more accurate (M = 98.0%, SD = 3.7) than those with DLD (M = 92.8%, SD = 6.6) on this verb generation task. However, the majority of children with DLD were able to perform this task well, see Fig. 2. Individuals categorised as HSL had an average accuracy of 94.4% (SD = 6.8).

## Planned analyses

### Evaluating hypothesis 1

**Fig. 3:**
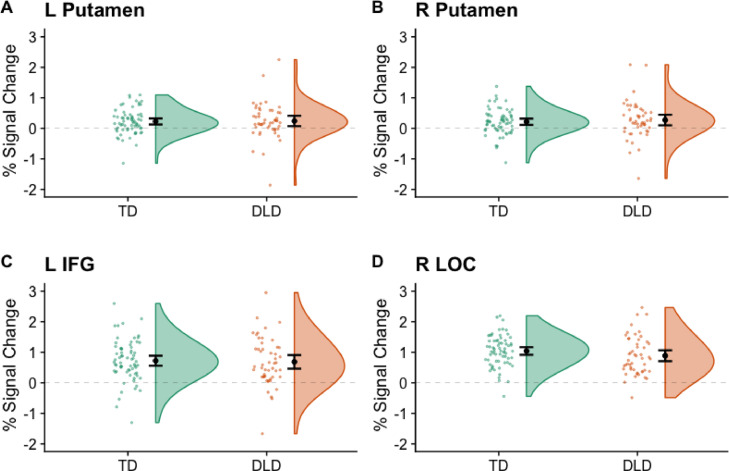
Percent BOLD signal change in TD (green) and DLD children (orange) in A) L Putamen, B) R Putamen, C) L Inferior Frontal Gyrus, pars triangularis, and D) R Lateral Occipital Cortex – plots show mean +/- 95% confidence intervals. Individual datapoints are shown alongside violin plots, with jitter added along the X dimension to aid visualisation.

We first tested hypotheses 1a-d, examining whether there were group differences in BOLD activity between TD children and those with DLD in 4 ROIs, the left putamen, the right putamen, the left inferior frontal gyrus and in a control region, the right lateral occipital cortex. We did not find significant differences in activity in any of these ROIs, see Table 2 and Fig. 3.

**Table 2:** Mean percent BOLD signal change during verb generation (SD). Group Differences in ROIs. P-values are not corrected for multiple comparisons.

ROI	TD	DLD	Statistics
L putamen	0.23 (0.41)	0.24 (0.59)	*t*(81.9) = .16, *p* = .87
R putamen	0.22 (0.42)	0.27 (0.61)	*t*(82.2) = .57, *p* = .57
L IFG	0.72 (0.67)	0.69 (0.79)	*t*(96.2) = .27, *p* = .79
R LOC	1.04 (0.51)	0.89 (0.63)	*t*(92.5) = 1.4, *p* = .16

We then conducted a set of regression analyses, in which the language and memory factors were used as predictors of activity in these four ROIs. This allowed us to evaluate if there was a continuous relationship between language/memory ability and activity in these four regions. We included the HSL participants in these analyses. We controlled for age and task performance in these models by entering them as control variables. Language, memory proficiency scores, age, or task accuracy did not significantly predict BOLD activity in any of our four ROIs of interest.

### Evaluating Hypothesis 2

**Fig. 4:**
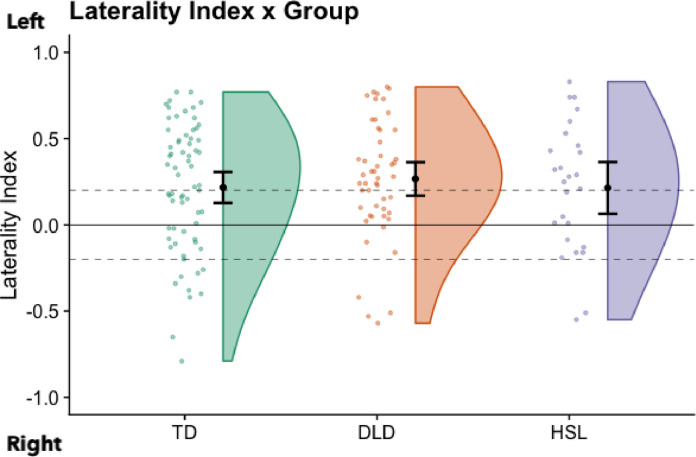
Laterality indices for verb generation evoked activity in the frontal lobes. Data for individual participants is shown (TD in green, DLD in orange, and HSL in purple). Black points show the mean for each group. Error bars represent +/- 95% confidence intervals. Laterality indices above the centre y = 0 line represent left lateralisation and values below this line represent right lateralisation. Additional lines have been placed at 0.2 and −0.2 as an indication of divisions for left, bilateral, and right lateralisation.

Laterality indices for this task indicated that most participants had left-lateralised activity for the verb generation task. In the typically developing group (N = 67), 36 children showed left-lateralised activity, 21 children had a bilateral pattern of activity, and 10 children exhibited right-lateralisation. In those with DLD, 32 were left-lateralised, 14 showed a bilateral pattern, and 4 were right-lateralised (N = 50). In the HSL group (N = 26), 15 were left-lateralised, 9 showed a bilateral pattern of activity, and 2 were right lateralised (see Fig. 4). To test hypothesis 2a, we assessed whether atypical lateralisation was associated with group. As the number of those with right-lateralised activity was less than 4 in the DLD group, we combined the right-lateralised and bilateral groups for the chi-squared analyses. These revealed that atypical lateralisation did not pattern with language status, X^2^ = 0.85, *p* = 0.36.

As above we conducted a regression analysis, in which the language and memory factors were used as predictors of the laterality indices including the HSL participants and controlling for age and task performance. Language or memory proficiency, age, or task accuracy did not predict LI values.

Given that we did not find evidence of reduced lateralisation in those with DLD, we did not test hypothesis 2b, i.e., that this difference might result from differences in noise levels.

## Exploratory analyses

### Whole-brain comparison of TD vs DLD children

**Fig. 5:**
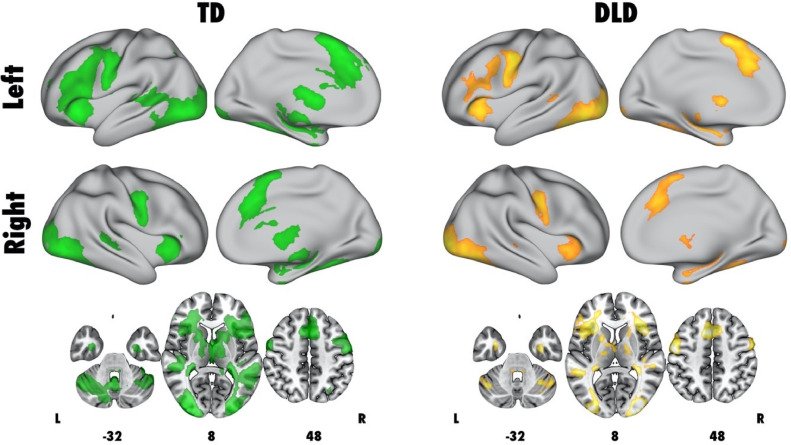
Group average BOLD activity during verb generation in TD (in green) and DLD children (in orange). Maps are displayed at a whole brain threshold of Z > 4.42.

While generating a verb corresponding to a picture, both TD children and those with DLD activated an expected and extended network of brain regions involved in speech and language processing. This included the left inferior frontal gyrus extensively, ventral sensorimotor cortex, supplementary motor complex (SMA and preSMA) extending ventrally to the cingulate cortex, and posterior superior temporal gyrus and sulcus bilaterally (see Fig. 5). Both groups also activated the occipital cortex bilaterally, associated with visual processing of the picture stimulus. In addition, we observed activity in sub-cortical regions such as the caudate nucleus and putamen. The anatomical location of statistical peaks, their MNI-space coordinates, z-statistics, and the extents of the cluster of voxels to which each is connected for the separate group analyses are presented in Table 3.

**Table 3:** Group average brain activity during verb generation > rest in A. typically developing children and B. children with DLD. Clusters with Z > 6 and a minimum extent of 50 voxels are reported in italics with up to 6 maxima per cluster. Peak locations are presented for X (sagittal), Y (coronal) and Z (axial) coordinates in mm relative to the orthogonal planes through the anterior commissure, together with peak z-statistic, and cluster extent in voxels. L, left; R, right.

Brain Area	X	Y	Z	Z-statistic	Voxels
**A. *TD***					
*Medial frontal cortex and right anterior insula*					4229
L anterior cingulate sulcus	-6	28	30	8.71	
R anterior cingulate sulcus	12	28	36	8.29	
R anterior insula	38	22	-6	8.74	
L pre supplementary motor area	-4	18	48	9.65	
*Left precentral gyrus extending to anterior insula*					5834
L anterior insula	-32	24	-4	9.37	
L ventral precentral gyrus	-50	-6	24	9.61	
L precentral gyrus (face representation)	-48	-12	36	9.46	
*Right precentral gyrus*					1144
R ventral precentral gyrus	58	-2	20	8.97	
R precentral gyrus (face representation)	48	-6	32	9.77	
*Right anterior parahippocampal gyrus*	30	-4	-36	7.48	52
*Thalamus and Brain stem*					618
R thalamus	14	-18	0	7.38	
R brainstem	12	-24	-12	9.63	
L brainstem	-12	-26	-12	9.08	
*Right superior temporal cortex*					277
R Heschl's gyrus	42	-22	10	7.20	
R posterior superior temporal sulcus	54	-28	4	8.26	
*Left superior temporal cortex (posterior)*	-56	-42	10	7.67	211
*Right Cerebellum*					403
R cerebellar lobule VIIb	32	-64	-50	8.16	
R cerebellar crus II	20	-76	-42	7.78	
*Occipital cortex (ventral)*					13056
R lateral occipital cortex	40	-74	-12	11.0	
L lateral occipital cortex	-34	-88	-10	11.0	
L occipital pole	-32	-90	-4	11.3	
R occipital pole	32	-92	-4	11.5	
**B. *DLD***					
*Left frontal opercular cortex*					524
L anterior insula	-32	24	0	7.37	
L frontal operculum (medial)	-38	12	16	7.31	
*Right frontal opercular cortex*					211
R anterior insula	40	18	0	7.04	
R frontal operculum (medial)	40	16	12	6.98	
*Medial frontal cortex*					1124
L paracingulate cortex	-4	18	44	7.60	
R cingulate sulcus	16	18	36	7.36	
L cingulate sulcus	-14	16	34	7.28	
L pre supplementary motor area	-6	4	60	8.04	
*Right precentral gyrus*					480
R ventral precentral gyrus	58	-2	20	6.57	
R precentral gyrus (face representation)	48	-10	36	8.04	
*Left precentral gyrus*					768
L ventral precentral gyrus	-56	-2	22	7.68	
L precentral gyrus (face representation)	-46	-14	40	8.45	
*Left occipito-temporal cortex*					2856
L mid fusiform gyrus	-38	-46	-8	9.55	
L lateral occipital cortex	-44	-72	-10	8.26	
L occipital pole	-26	-90	-6	8.88	
*Right occipital cortex (ventral)*					3024
R mid fusiform gyrus	34	-48	-18	8.49	
R cerebellar lobule VI	16	-60	-20	8.47	
R lateral occipital cortex	40	-86	-8	8.88	
R occipital pole	32	-90	-2	9.67	

We examined whether there were TD vs. DLD group differences in brain activity in regions other than those assessed in the ROI analyses above. No clusters survived thresholding at Z > 3.1 with a cluster-forming threshold of *p* < 0.05. We lowered the threshold (at Z > 2.3 with a cluster-forming extent of 50 voxels) to explore the potential for false negatives; at this lower threshold, children with DLD showed greater activity than those who were TD in the right angular and supramarginal gyri. These regions were not robustly activated during task performance, and appeared to be at the brain boundary, decreasing our confidence in these results. TD children showed greater activity than those with DLD in the occipital cortex and anterior cingulate cortex (see Table 4). Again, the cluster in the anterior cingulate was not active during task performance. The clusters in occipital cortex were unexpected and contrary to previous studies as well as our hypotheses, and not part of the core language network. Overall, there were no robust differences in language task-evoked activity in children with DLD compared with TD children. This lack of group differences is consistent with the negative results of the planned ROI analyses.

**Table 4:** Group Differences in activity during Verb Generation. Contrast images for groups were thresholded at Z > 2.3 (uncorrected). Peak coordinates for clusters with a minimum extent of 50 voxels are reported. See legend to Table 3 for details.

Brain Area	X	Y	Z	Z-statistic	Voxels
***TD > DLD***					
L superior frontal gyrus	-2	52	48	3.29	181
R rostral gyrus / anterior cingulate cortex	4	34	-8	3.35	76
R mammillary body / nucleus accumbens	4	-2	-10	3.49	150
L lateral occipital cortex	-26	-90	6	3.83	428
R lateral occipital cortex	20	-90	-8	4.03	905
L occipital pole	-4	-90	-8	4.00	399
***DLD > TD***					
R middle frontal gyrus	42	16	60	3.40	79
R globus pallidum	22	-12	6	3.41	60
R supramarginal gyrus	62	-34	50	3.23	55
R angular gyrus	48	-56	58	3.26	84

### Whole brain correlation analysis

**Fig. 6:**
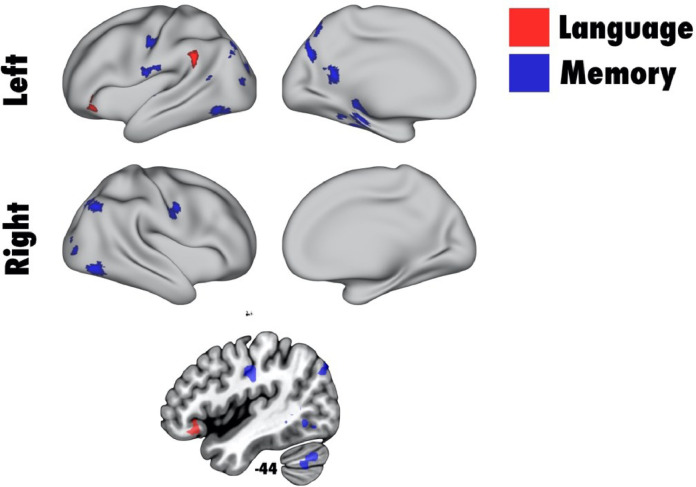
Brain areas showing positive correlations between brain activity during verb generation and language proficiency (in red) or verbal memory ability (in blue). Maps are thresholded at Z > 2.3 (uncorrected), with a minimum cluster extent of 50 voxels. A sagittal slice in included to show the cluster in the cerebellum.

As planned, we conducted a correlation analysis using the summary measures for language and memory functions described above and neural activity for the verb generation > rest contrast across the entire cohort/population of children with eligible data (N = 143). This allowed us to assess how variation in language and memory ability patterned with brain activity for verb generation. We did not observe any correlations when using a threshold of Z > 3.1, with a cluster correction of *p* < .05. However, on lowering the threshold (Z > 2.3, with a cluster-forming extent of 50 voxels) we observed that higher language proficiency was associated with greater task-related activity in the left inferior frontal gyrus (pars orbitalis) and the left supramarginal gyrus (Fig. 6). Importantly, both these regions were activated during task performance. Better verbal memory ability was associated with greater task-related activity in a range of areas, including the left cerebellum and ventral sensorimotor cortex bilaterally (Fig. 6). The anatomical location of statistical peaks, their MNI-space coordinates, z-statistics, and the extents of the cluster of voxels containing these peaks are presented in Table 5.

**Table 5:** Whole-brain correlation analysis with language and memory factors. Clusters showing positive correlations with Z > 2.3 (uncorrected) and minimum extents of 50 voxels are reported in italics with the locations of up to 6 maxima per cluster. See Table 3 for details.

Brain Area	X	Y	Z	Z-statistic	Voxels

***Language Proficiency***					
*Left inferior frontal gyrus (orbitalis)*	-48	30	-12	2.97	61
*Left supramarginal gyrus*	-60	-46	28	3.23	95
***Verbal Memory Ability***					
*Right postcentral gyrus (face representation)*	50	-12	34	3.2	85
*Left postcentral gyrus (face representation)*	-42	-18	32	3.49	94
*Left parieto-temporal operculum*					90
L planum temporale	-70	-18	8	3.28	
L parietal operculum	-62	-12	16	2.78	
*Left posterior medial temporal cortex*					480
L posterior hippocampus	-30	-34	-2	3.55	
L parahippocampal gyrus (posterior)	-24	-32	-16	3.5	
L fusiform gyrus (anterior)	-32	-32	-24	3.17	
*Brainstem*	6	-40	-40	3.36	76
*Left retrosplenial cortex*	-6	-54	10	3.00	61
*Left lateral occipital cortex*	-42	-62	-6	3.01	60
*Left cerebellum*					474
L cerebellum, Crus II	-32	-64	-44	4.13	
L cerebellum, Crus I	-42	-64	-34	3.17	
*Left superior parietal lobule*	-10	-68	56	3.15	54
*Right lateral occipital cortex*	46	-70	-10	3.05	111
*Right precuneous cortex*	-12	-70	32	2.82	52
*Right posterior parietal cortex*					419
R superior parietal lobule	16	-76	58	3.43	
R inferior parietal lobule	36	-72	48	3.30	
*Left inferior parietal lobule*	-32	-82	44	4.01	244
*Right lateral occipital cortex*	36	-84	6	2.98	54

### Variability analyses

**Fig. 7:**
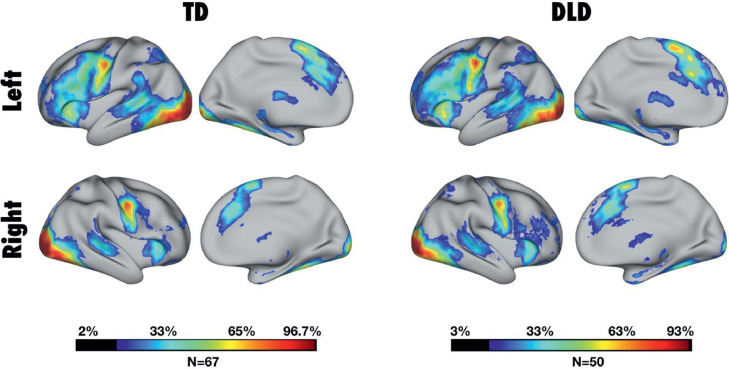
Probabilistic overlap maps showing consistently activated regions in children who were TD or DLD. Colours represent the percentage of participants that activate a specific area, with hot colours showing the greatest overlap and cool colours showing relatively limited overlap.

The whole-brain averages of task-evoked activity discussed above may not fully reflect inter-subject variability (see Olulade et al., 2020 for a discussion of similar issues), which we expected might be greater in those with DLD. To visualise consistency in patterns of activation across TD and DLD, we generated probabilistic overlap maps. These were generated by summing up individual activity maps created using Z > 4.42 in the TD and DLD participants. In these maps, voxels that are consistently activated by the majority of participants can be easily identified, which enables comparisons of variability across groups. Our groups differed in the number of children (TD N = 67; DLD N = 50). To facilitate comparison across groups, we converted these maps to reflect the percentage of children in the group who activated a specific area. These probabilistic overlap maps indicated “hotspots” of activity in left inferior frontal gyrus, motor cortex at the level of the face, superior temporal gyrus and occipital regions bilaterally in neurotypical children (Fig. 7). The overlap map for DLD bore close resemblance to the map for typically developing children. The only obvious minor difference was that a few participants with DLD (<30%) showed some task-related activity in the right inferior frontal gyrus extending dorsally, whereas very few TD participants activated this region (Fig. 7, right panel).

## Unplanned analyses

### Groups compared based on task performance

**Fig. 8:**
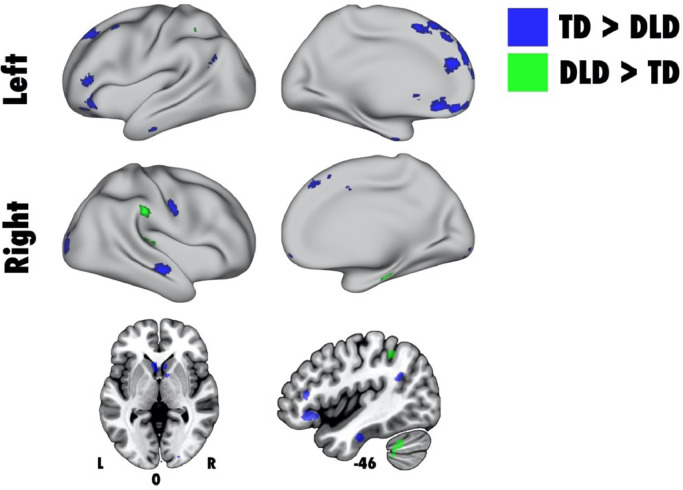
Results of the performance sub-group analysis. Regions indicated in blue are those in which high-performing typically developing children show greater activity than low-performing children with DLD, and regions in green are those in which children with DLD show greater activity relative to TD children. Maps are thresholded at Z > 2.3, with a minimum cluster extent of 25 voxels. A sagittal slice is included to show the cluster in the cerebellum, and an axial slice is included to show differences in the caudate nuclei.

Our task is one of the first to control for performance, in that we overtly assessed accuracy, and only analysed data from children who could perform the task fairly well. To assess how previous studies that compared DLD and TD children may have been affected by differences in task performance, we selected a subset of the DLD group with the lowest verb generation accuracy, i.e., the lowest quartile (50–83.3% accuracy, N = 14). This included the 4 children with DLD we previously excluded from analyses due to low accuracy (<75%). We compared this group to a subset of TD children matched for age and gender, but who performed the task with high accuracy (100%, N = 14). No clusters survived thresholding at Z > 3.1 with an extent threshold of *p* < 0.05 (corrected). We lowered the threshold to at Z > 2.3 with an extent threshold of 25 voxels (uncorrected) to explore the potential of false negatives, especially given the smaller sample. At this threshold, several areas showed reduced activity in the low-performing DLD group relative to the TD group, including in the left IFG (pars triangularis) extending into frontal orbital cortex, and in caudate nuclei bilaterally (see Supplementary Table 1 and Fig. 8). This was consistent with previous studies (including our own) of small samples where task performance was uncontrolled. In the right parahippocampal gyrus, left postcentral gyri, left cerebellum, brain stem, and in small clusters in the supramarginal gyri bilaterally, the low-performing DLD group showed increased activity relative to TD children.

## Discussion

In this large study comparing children with developmental language disorder (DLD) to typically developing (TD) children, we tested for differences in brain activity for verb generation. We found little support for our two key hypotheses. First, we failed to show group differences in task-related activity in the left inferior frontal gyrus and putamen bilaterally (see Fig. 3). Second, we did not show any evidence for atypical lateralisation in those with DLD (see Fig. 4). As highlighted previously, the few fMRI studies that have examined functional activity in children with DLD used a variety of tasks and yielded findings that are inconsistent with each other (Mayes et al., 2015). In addition, most of these studies did not capture performance, or they used tasks in which children with DLD perform poorly. We believe this study fills an important gap in the functional imaging literature on DLD, addressing concerns of small sample sizes and population heterogeneity. Our findings, obtained in the largest sample of children with DLD studied so far, indicate that when using a task that children with DLD can perform, they activate the same brain regions as those who are typically developing, and to a similar extent. Regions in the brain that are sensitive to variation in language and verbal memory ability were revealed, however, by our analysis correlating continuous measures of these factors with task-related activity for verb generation. This is a very useful starting point in our understanding of the brain basis of DLD. Importantly, our work emphasises the need to control for task performance. As we discuss below, it also suggests we will need to tap into more complex language constructs to find neural differences in this group.

### Lack of frontostriatal dysfunction for verb generation in children with DLD

In this well-powered study, we tested for group differences in activity in key regions of the frontostriatal network, such as the putamen and the left inferior frontal gyrus – but did not find any evidence for such differences. Our conclusion was supported by our continuous analyses, as differences in brain activity in these regions were not accounted for by our measures of language or memory functioning, or age, or accuracy. Our findings indicate a lack of support for the procedural deficit hypothesis (Ullman and Pierpont, 2005), and our own theory suggesting corticostriatal functional abnormalities in DLD (Krishnan et al., 2016); those with language disorders do not appear to show abnormal function in two important regions of the frontostriatal loop, namely the left inferior frontal gyrus and the putamen, during a simple verb generation task. Sub-threshold differences in the caudate nuclei bilaterally and left inferior frontal gyrus were revealed when we focused on the children with DLD who had very low verb generation task performance in the scanner (see Fig. 8). In our view, this exploratory analysis illustrates that striatal differences reported in previous studies most likely reflect differences in task performance, rather than being characteristic of functional neural differences in DLD *per se*. Indeed, we believe the results of this small group emphasise why large sample sizes, and careful monitoring of performance, are necessary when conducting imaging studies of children with DLD.

However, we believe it would be premature to conclude that frontostriatal regions function normally in those with DLD on the basis of this one task. First, the nature of our task, which was designed to be simple and easy to perform, may have reduced our sensitivity to detect differences in these regions. Previous studies have suggested that striatal regions are activated by difficult or novel articulatory-phonological processing (Klein et al., 2006; Simmonds et al., 2014). Tasks that involve sequential learning, such as learning the form of a novel word, may be better suited to revealing differences in these regions. (but note that Pigdon et al., 2020 reported no differences in brain activity for nonword repetition in children with DLD). Our task was also very short; it is possible that having more trials and consequently better estimates of activity would reveal more subtle differences (however, the trade-off here is that children are less likely to tolerate longer tasks and more likely to move). Second, there may be a limited time window in development during which these influences are seen; a recent review suggests that the contribution of the striatal circuit to speech motor learning may be confined to the period of articulatory skill acquisition (Ziegler and Ackermann, 2017). In this study, we tested children and adolescents between the ages of 10 and 15 years. Younger children with DLD, who are in the process of learning words and refining their speech production system, may differently activate frontostriatal regions. Third, more detailed analyses may reveal differences in connectivity or microstructure of the striatum. For instance, recent studies suggest that profiles of brain connectivity of specific hub regions are associated with cognitive profiles (Lee et al., 2020; Siugzdaite et al., 2020). Despite these caveats, a key takeaway from our data is that functional differences in these regions in adolescence are, if present, likely to be subtle.

### Lack of evidence for atypical frontal lateralisation in DLD

We also assessed differences in frontal lateralisation for verb generation. We did not find any evidence that left-lateralisation was less common in those with DLD or in those with a history of speech and language disorders. This is consistent with findings from recent studies using functional transcranial Doppler (Wilson and Bishop, 2018). One criticism of fTCD is that it might not be sensitive to regional differences in lateralisation, which fMRI is better suited to assess. However, our results, especially when taken in conjunction with fTCD findings, provide convincing evidence that previously reported differences in lateralisation are likely to have been false positives.

### Lack of group differences beyond the frontostriatal network

Our verb generation task evoked activity in both DLD and TD groups in the inferior frontal gyrus, the superior temporal gyrus, primary motor cortex at the level of the articulators, supplementary motor areas and occipital cortex bilaterally (see Fig. 5), which is consistent with previous reports of brain activity for this task. We examined if there were group differences in activity in this broader language network. A whole-brain analysis did not reveal any group differences in the language network between those with DLD and neurotypical children when conventional thresholds (cluster-forming Z > 3.1, extent *p* < 0.05) were used. Although some differences can be observed when lowering the threshold, these are not in the areas where we predicted differences, and we believe that a threshold of Z > 3.1 is appropriate when considering our sample size (interested readers can independently assess these maps at https://identifiers.org/neurovault.collection:8615). Our negative findings at the group level are consistent with those from a recent study of DLD and developmental speech sound disorder, which also suggested these groups had similar patterns and levels of brain activity to those seen in typically developing children (Pigdon et al., 2020).

### Is the lack of functional neural differences due to greater neural variability in children with DLD?

We theorised that those with DLD might be more variable as a group in terms of the regions they activated, relative to typically developing children. Such neural heterogeneity has been shown in other groups with neurodevelopmental disorders such as autism (Hahamy et al., 2015), and has been postulated for disorders such as developmental dyslexia (Hancock et al., 2017). To assess functional heterogeneity, we examined overlap maps to assess the regions that children strongly activated at an individual level while completing verb generation tasks (see Fig. 7). These indicate that both typically developing children and children with DLD activated the motor regions of cortex representing the articulators and visual cortex very consistently. Activation of voxels in the left inferior frontal gyrus showed somewhat lower consistency spatially across participants but are clearly activated during this task by both groups. There is relatively little evidence for consistent activation of the putamen (Supplementary Figure 1). Examination of these overlap maps provides further support for the lack of group differences in brain activity for this task, suggesting that the task evokes activity in very similar brain networks in both groups to similar extents.

### Moving beyond group dichotomies to continuous measures of language variation

In the aforementioned analyses, we first used a categorical approach, testing differences in brain activity only in children with DLD and those who were typically developing. We then used a continuous approach, deriving language proficiency scores for DLD, TD and HSL groups. Our data reduction was planned specifically to circumvent having multiple scores from each of our behavioural tests to correlate with the imaging data. One of the issues with this practice is that it is very easy to then deliver explanations for why each individual measure was well-suited to capturing language variation (i.e. HARKing, see Bishop, 2019; Bishop, 2020). We found that the variance in our behavioural data was best captured by calculating two factors (language and memory). We anticipated that our continuous approach would be more sensitive to variation in brain activity than extreme group analyses, as we could use data from children who had a history of speech and language problems, and we were not limited to coarse group comparisons. While this approach yielded similar results within our pre-defined ROIs and in the laterality analysis, the whole-brain correlations did yield some interesting findings (see Fig. 6).

Specifically, our language factor was associated with increases in activity in the left inferior frontal gyrus (pars orbitalis) and the left supramarginal gyrus. These regions, and the supramarginal gyrus/ inferior parietal cortex in particular, have been linked to phonological working memory (Baldo and Dronkers, 2006; Friederici, 2012; Paulesu et al., 1993), and might be engaged in this task to temporarily store or rehearse phonological information. The region in the supramarginal gyrus that we find is also close to area Spt, which has been postulated to be a sensory-motor interface, supporting interactions between articulatory and temporal regions during phonological processing, and playing a key role in verbal working memory (Buchsbaum et al., 2001; Hickok et al., 2003). Alternatively or in addition, performance correlations with activity in the left inferior frontal gyrus could reflect selection demands during verb generation (Thompson-Schill et al., 1997). Accordingly, with larger vocabularies or greater language skills, selection demands of the verb generation task might be higher, leading to greater activity in this region in more proficient participants. Our memory factor was positively associated with task-related activity as expected in the left medial temporal cortex, including the hippocampus. It was also positively correlated with activity in ventral sensorimotor cortex bilaterally, and the cerebellum. These regions are linked to speech production, and their engagement in this task may suggest more efficient retrieval of articulatory plans for speech, which was required for many of the verbal memory tasks. Supporting this interpretation, a previous study has shown that changes in verbal IQ are linked to structural change in ventral sensorimotor cortex (Ramsden et al., 2011). Taken together, these findings suggest that differences in aspects of language ability are reflected in brain activity for a language task, with specifics of performance linked to increased activity in specific nodes of the language network. This points to a lack of sharp dividing lines between TD and DLD brains, especially for tasks that children can perform. Modelling continuous language variation in large samples might yield greater insight into the brain basis of DLD than using dichotomous categories. We stress, however, that these findings are exploratory, and will need replication.

### Summary and conclusions

In summary, we find little support for frontostriatal dysfunction in brain activity, or atypical lateralisation, during a verb generation task in children with DLD. This might be because our task made few demands on language learning or complex language processing. Even so, this is important evidence to suggest that for simple tasks where performance is controlled, those with DLD show brain activity that resembles that of typically developing children. To probe why language difficulties are observed in DLD, we now need to either use more sensitive measures of brain activity and connectivity, or design tasks that tap specific aspects of language that are affected in children with DLD, for instance, sequential learning. Our results also yield some promising directions for future study; specifically, we observe a relationship between distinct cortical brain regions and our language and verbal memory factors. This indicates that sub-groups of those with DLD with poor language or poor memory may recruit these regions less efficiently.

## Data availability

Anonymised neuropsychological scores, as well as code to run the verb generation task and the factor analysis, are available on Open Science Framework (https://doi.org/10.17605/OSF.IO/2WPX5). Anonymised raw MRI data (T1 weighted scans and fMRI data) are openly available for download via OpenNeuro (https://openneuro.org/datasets/ds003145).

## Appendix Supplementary materials

Image, application 1
