# Supplementary material for: Functional organisation for verb generation in children with developmental language disorder
Source: Neuroimage. 2021 Feb 1;226:117599. doi: 10.1016/j.neuroimage.2020.117599 (PMC7836232; doi:10.1016/j.neuroimage.2020.117599)
Supplement: Supplementary file 1 [file mmc1.docx]

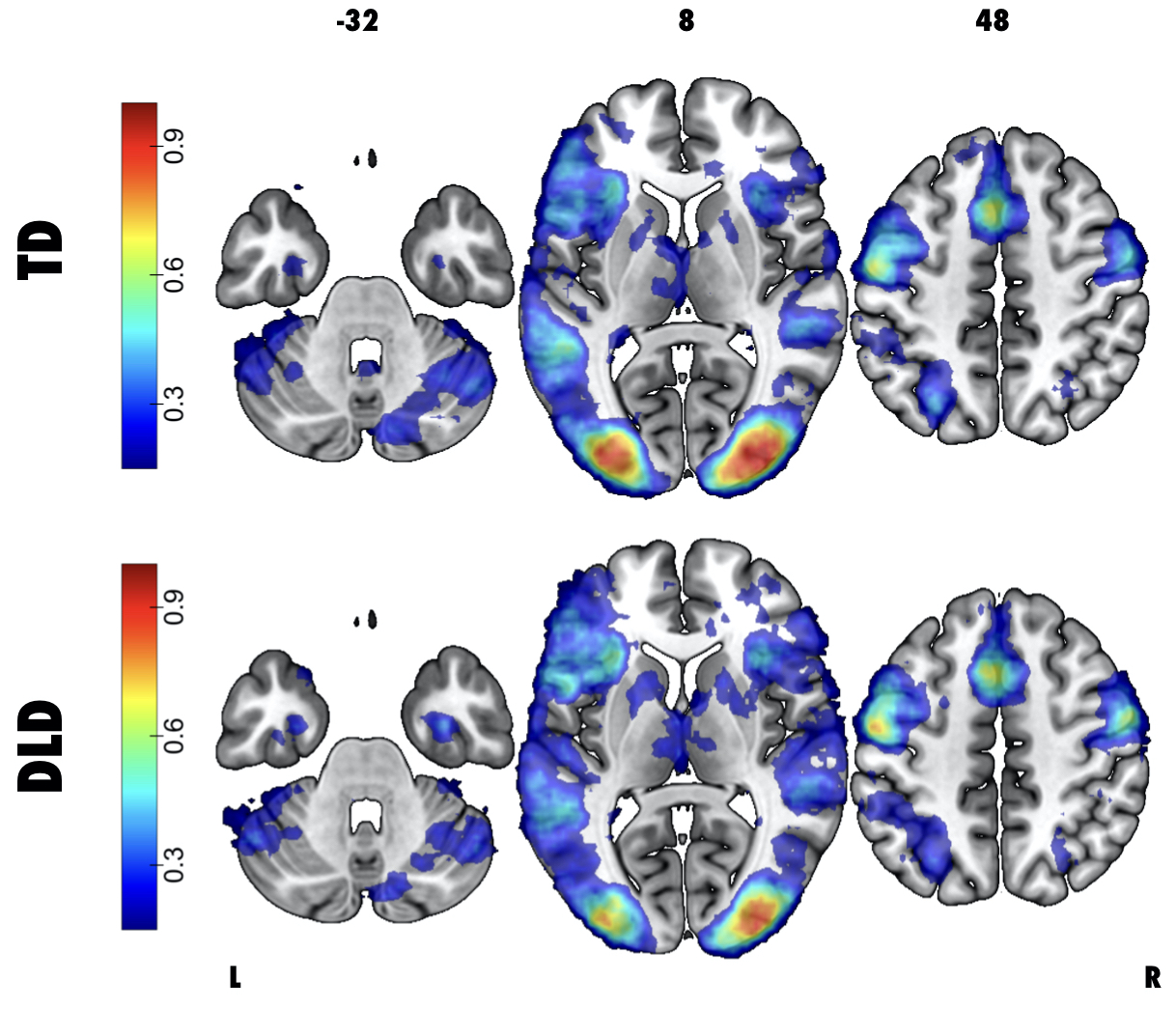


*Supplementary figure 1: Probabilistic overlap maps in TD and DLD children, showing the low consistency of activation in subcortical regions and in the cerebellum. Colours represent the percentage of participants that activate a specific area, with hot colours showing the greatest overlap and cool colours showing relatively limited overlap.*

Supplementary Table 1. Results the performance sub-group analysis, capturing group-differences in brain activity between the DLD children with the lowest accuracy and the TD children with the highest accuracy. Differences are significant at Z > 2.3 and with extents of 25 or more voxels. Brain locations are presented for X (sagittal), Y (coronal) and Z (axial) coordinates in mm relative to the orthogonal planes through the anterior commissure, together with peak z-statistic, and extent size in voxels.

| Brain Area | X | Y | Z | Z-statistic | Voxels |
| --- | --- | --- | --- | --- | --- |
| ***TD>DLD*** | | | | | |
| L orbitofrontal cortex | -36 | 40 | -12 | 3.46 | 30 |
| L anterior paracingulate gyrus | -10 | 38 | 22 | 2.96 | 41 |
| R superior frontal gyrus (medial) | 8 | 34 | 48 | 3.52 | 45 |
| L inferior frontal gyrus, pars triangularis | -40 | 34 | 10 | 3.64 | 47 |
| L subcallosal / orbitofrontal cortex | -8 | 32 | -12 | 3.09 | 186 |
| L superior frontal gyrus (medial) | -18 | 32 | 48 | 3.61 | 403 |
| L inferior frontal gyrus, pars orbitalis | -44 | 28 | -8 | 3.24 | 110 |
| Left caudate nucleus | -6 | 18 | 0 | 3.26 | 41 |
| Right caudate nucleus | 6 | 16 | 0 | 2.86 | 30 |
| L middle frontal gyrus | -38 | 14 | 54 | 3.11 | 33 |
| L presupplementary motor area | -6 | 12 | 50 | 3.18 | 65 |
| L entorhinal cortex | -28 | -6 | -38 | 3.25 | 38 |
| R central sulcus (face representation) | 44 | -8 | 30 | 3.67 | 67 |
| L inferior temporal gyrus | -46 | -14 | -28 | 3.46 | 42 |
| R superior temporal sulcus | 46 | -18 | -14 | 3.37 | 94 |
| L parahippocampal gyrus | -32 | -24 | -28 | 3.92 | 33 |
| L angular gyrus | -36 | -52 | 30 | 3.57 | 129 |
| R occipital fusiform gyrus | 34 | -80 | -18 | 2.8 | 34 |
| R occipital pole | 26 | -88 | 10 | 3.02 | 74 |
| ***DLD > TD*** | | | | | |
| R parahippocampal gyrus | 18 | -24 | -20 | 3.31 | 27 |
| Brainstem | 4 | -26 | -26 | 3.47 | 102 |
| R planum temporale (medial) | 34 | -30 | 10 | 3.42 | 65 |
| R supramarginal gyrus | 58 | -30 | 34 | 2.93 | 31 |
| L postcentral gyrus | -52 | -36 | 58 | 2.85 | 26 |
| L supramarginal gyrus | -44 | -46 | 48 | 2.77 | 30 |
| L cerebellum, Crus I | -46 | -52 | -38 | 3.23 | 74 |
| L superior parietal lobule | -4 | -58 | 72 | 3.60 | 68 |
|  |  |  |  |  |  |
|  |  |  |  |  |  |
